# Mechanistic role of mesencephalic astrocyte-derived neurotrophic factor in myocardial ischemia/reperfusion injury

**DOI:** 10.1186/s10020-024-00927-3

**Published:** 2024-10-26

**Authors:** Fahimeh Varzideh, Brandon Wang, Yifei Qin, Urna Kansakar, Gaetano Santulli, Stanislovas S. Jankauskas

**Affiliations:** 1 Department of Medicine, Division of Cardiology, Wilf Family Cardiovascular Research Institute, Einstein Institute for Neuroimmunology and Inflammation (INI), Albert Einstein College of Medicine, Montefiore University Hospital, 1300 Morris Park Avenue, New York, NY 10461 USA; 2 Department of Molecular Pharmacology, Einstein-Mount Sinai Diabetes Research Center (ES-DRC), Fleischer Institute for Diabetes and Metabolism (FIDAM), Einstein Institute for Aging Research, Albert Einstein College of Medicine, Montefiore University Hospital, New York, NY 10461 USA

## Abstract

Mesencephalic astrocyte-derived neurotrophic factor (MANF) is a protein crucial for cellular stress response and survival, particularly in the nervous and cardiovascular systems. Unlike traditional neurotrophic factors, MANF primarily regulates endoplasmic reticulum (ER) stress and protects cells by reducing ER stress-induced apoptosis. MANF operates both inside and outside cells, influencing key pathways like JAK/STAT and NF-κB to enhance cell survival during stress. Beyond its neuroprotective role, MANF is also vital in cardiovascular protection, mitigating damage by reducing inflammation and maintaining cellular function. Elevated MANF levels have been observed in patients experiencing myocardial infarction and murine models of ischemia–reperfusion (I/R) injury, highlighting its importance in these conditions. Overexpression of MANF in cardiomyocytes reduces ER-stress-induced cell death, while its depletion worsens this effect. Treatment with recombinant MANF (rMANF) has been shown to improve cardiac function in mice with I/R injury by decreasing infarct size and inflammation. Research also indicates that alterations in the α1-helix region of MANF can impact its structure, expression, secretion, and overall function. Given its protective effects and involvement in critical signaling pathways, MANF is being explored as a potential therapeutic target for ER stress-related diseases, including neurodegenerative disorders and cardiovascular conditions like myocardial I/R injury.

Mesencephalic astrocyte-derived neurotrophic factor (MANF), also known as Arginine-rich, mutated in early-stage tumors (ARMET), or Arginine-rich protein (ARP), is a protein that plays a vital role in cellular stress response and survival, particularly within the nervous and cardiovascular systems (Danilova et al. 2019). Unlike traditional neurotrophic factors, MANF is primarily involved in the regulation of endoplasmic reticulum (ER) stress and has been shown to exert protective effects by alleviating ER stress-induced apoptosis. MANF functions both intracellularly and extracellularly, modulating key signaling pathways, including the JAK/STAT and NF-κB pathways, to promote cell survival under stressful conditions. In addition to its neuroprotective properties, MANF has been implicated in cardiovascular protection, where it helps mitigate damage by reducing inflammation and preserving cellular function. Ongoing research is exploring its therapeutic potential in treating a wide range of ER stress-related diseases, including neurodegenerative disorders and cardiovascular conditions, including myocardial ischemia–reperfusion (I/R) injury, which is a serious and potentially lethal condition that ranks among the leading causes of death globally.

Myocardial I/R injury refers to the tissue damage that occurs when blood supply returns to the heart after a period of ischemia, or reduced blood flow (Hausenloy and Yellon 2013). Although restoring blood flow is essential for salvaging heart tissue and preventing infarction, the sudden reoxygenation and influx of nutrients paradoxically cause a cascade of biochemical and cellular events that can exacerbate tissue injury. During the ischemic phase, a lack of oxygen and nutrients leads to the accumulation of metabolic waste products and a shift to anaerobic metabolism, resulting in acidosis, depletion of ATP, and cellular dysfunction (Kalogeris et al. 2012; Zong et al. 2024). Upon reperfusion, the reintroduction of oxygen triggers the production of reactive oxygen species (ROS), leading to oxidative stress, lipid peroxidation, and damage to cellular membranes, proteins, and DNA (Marti-Pamies et al. 2023). Additionally, reperfusion causes a rapid influx of calcium ions into cardiomyocytes, which can overload the mitochondria (Santulli et al. 2015a) and further contribute to cell death through apoptosis or necrosis (Re et al. 2019). Inflammatory responses are also activated, with neutrophils and other immune cells infiltrating the myocardium, releasing cytokines and other mediators that exacerbate tissue damage (Pluijmert et al. 2021; Saxena et al. 2015).

Collectively, these processes can result in significant myocardial injury, which manifests clinically as reduced cardiac function, arrhythmias, and heart failure. Myocardial I/R injury is a critical factor in the outcomes of patients undergoing treatments such as thrombolysis or percutaneous coronary interventions (PCI) for acute myocardial infarction. Understanding and mitigating I/R injury is therefore a major focus of cardiovascular research. In the current issue of *Molecular Medicine*, Dong, Jia and colleagues (Dong et al. 2024) observed increased levels of MANF protein in both myocardial infarction (MI) patients and mice undergoing I/R injury, underscoring the crucial involvement of MANF in these pathological processes. Overexpression of MANF in cardiomyocytes was found to reduce ER-stress-induced cell death, while depletion of MANF aggravated this condition. Treatment with recombinant MANF (rMANF) was shown to improve cardiac function in mice with I/R injury by reducing infarct size and inflammation. Additionally, the study suggests that alterations in the α_1_-helix region of MANF (Fig. 1) might affect its structure, expression, secretion, and overall function.

**Fig. 1:**
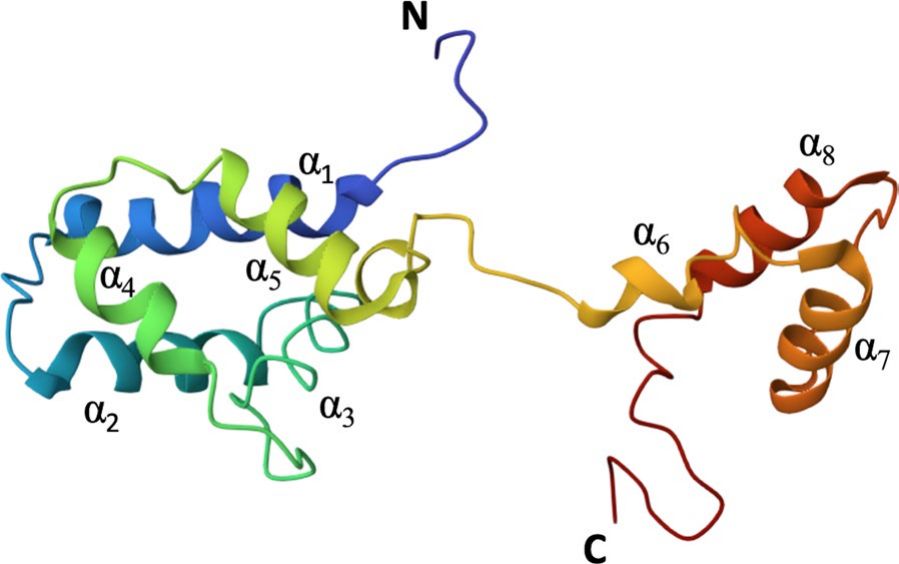
Structure of mesencephalic astrocyte-derived neurotrophic factor (MANF) showing its eight helices

Various studies have shown that MANF expression increases in different conditions associated with ER stress, including cancer, glomerular disease, rheumatoid arthritis, liver damage, and systemic lupus erythematosus (Wang et al. 2021; Xiong et al. 2024; Qin et al. 2024; Xie et al. 2024; Huang et al. 2024; Yu et al. 2024; Zhao et al. 2024). Similarly, Dong, Jia and colleagues detected an augmented release of MANF following ER stress induced by ischemia: elevated MANF levels were observed in HL-1 cells following hypoxia/reoxygenation (H/R) stimulation, and increased MANF levels were also found in the serum of MI patients and I/R mice (Dong et al. 2024). Strikingly, pre-ischemia administration of rMANF improved myocardial function and reduced infarct size, highlighting MANF's protective role as an ER stress-induced secreted cardiomyokine.

The α-helix is a common secondary structure in proteins, critical for their stability and architecture; mutations can disrupt this helical structure, impairing proper protein folding and maintenance of natural conformation (Eisenberg 2003). However, mutations can sometimes result in new protein functions by altering the overall structure to interact with different molecules or participate in novel biological processes. Given the distinct folding conformation of MANF (Yan et al. 2019), a series of mutants (M1-M8) were generated by inserting proline into the corresponding α-helix to explore the influence of the α-helix on MANF's secretion and functionality. Immunoblot analyses following the overexpression of these mutants revealed that two critical α-helices (α_1_ and α_7_) in the secondary structure of MANF significantly impacted its intracellular transport and secretion. Specifically, the M1 mutant exhibited primarily intracellular expression with minimal extracellular secretion, while the M7 mutant displayed a decreased intracellular expression but elevated secretion levels in comparison to the wild-type MANF. Furthermore, the M1 mutant provided greater protection against myocardial injury under H/R conditions (Dong et al. 2024), suggesting that retaining M1 in the intracellular ER following the α1-helix mutation might enhance the cytoprotective function of MANF under stress conditions. Hence, α1-helix mutations not only affect the structure and localization of MANF but also bolster its protective capabilities.

Under ischemic and hypoxic conditions, intrinsic cellular mechanisms respond to ER stress by activating the unfolded protein response (UPR) signaling pathway (Meyer and Doroudgar 2020; Mariangelo et al. 2020). This process involves three ER transmembrane protein receptors: IRE1α, PERK, and ATF6 (Keestra-Gounder et al. 2016; Le and Kimata 2021; Santulli et al. 2015b). PERK, with its kinase activity, phosphorylates eIF2α, leading to a selective inhibition of overall protein synthesis and retention of the transcription factor ATF4, which then translocates to the nucleus to regulate the transcription of UPR-related genes. Additionally, PERK activation induces CHOP-mediated apoptosis. The overexpression of MANF resulted in decreased levels of UPR-related proteins such as ATF6, p-PERK, p-IRE1α, BiP, p-eIF2α, CHOP, and ATF4 in cells. The ability of M1 to reduce the expression of these proteins was more pronounced compared to wild-type and M7 (Dong et al. 2024), suggesting that MANF and M1 mitigate apoptosis by inhibiting UPR signaling activation and alleviating H/R-induced ER stress. Furthermore, all three UPR sensors are activated to some extent by H/R, indicating a potential interconnection between the UPR branches in the context of cardiac H/R injury. MANF and M1 were found to exert a protective effect against H/R-induced cell injury by reducing apoptosis through ER stress mitigation. Notably, the mutation of the α-helix structure in M1 enhanced its ability to counteract oxidative stress, warranting further investigation into the precise mechanism by which MANF modulates cellular stress responses.

The primary mechanism through which MANF protects the heart from I/R injury is by attenuating ER stress, but additional mechanisms are also involved. Mounting evidence indicates that the JAK1/STAT1 signaling pathway is implicated in apoptosis (Hu et al. 2021; Seif et al. 2017). Cytokine binding to receptors triggers receptor dimerization, leading to JAK activation and subsequent phosphorylation of STAT family members. This cascade is crucial in stress signaling pathways governing gene expression and cell death (Kishore and Verma 2012). Several studies have indicated that the JAK1/STAT1 signaling pathway modulates inflammation and left ventricular remodeling post-myocardial infarction (Zhang et al. 2022, 2023), thereby mitigating myocardial I/R injury in cardiac tissues and cardiomyocytes. Such a pathway can reduce cardiac hypertrophy by enhancing autophagy and inhibiting mitochondria-dependent apoptosis (McCormick et al. 2012; Liu et al. 2023). These findings suggest a pivotal role of the JAK1/STAT1 pathway in the regulation of cellular inflammation and apoptosis during I/R injury. In their study, Dong, Jia and collaborators demonstrated via in vitro experiments that H/R significantly increased the levels of p-JAK1 and p-STAT1, while overexpression of MANF and M1 reduced their expression in myocardial I/R. Similar to its ER stress attenuation ability, M1 showed a more pronounced decrease in p-JAK1 and p-STAT1 levels compared to wild-type and M7. In vivo experiments similarly concluded that rMANF effectively reduces p-JAK1 and p-STAT1 levels. The consistency between in vivo and in vitro experimental results enhances the credibility of these conclusions.

Crystal structure analyses of MANF (Fig. 1) revealed that its C-terminal structural domain is similar to Ku 70, an anti-apoptotic protein (Amsel et al. 2008) that interacts with pro-apoptotic Bax (Amgalan et al. 2020) and is homologous to its structural domain SAP (named after SAF-A/B, Acinus and PIAS, three proteins known to contain it), a 35-residue motif found in a variety of nuclear proteins involved in transcription, DNA repair, RNA processing or apoptotic chromatin degradation. SAP-like structural domains have been shown to promote MANF interaction with p65 and negatively regulate NF-κB signaling under inflammation and ER stress (Yu et al. 2021; Tang et al. 2022).

Thus, MANF can inhibit the JAK1/STAT1/NF-κB signaling pathway; however, further research is needed to explore potential additional connections within this signaling pathway. These findings emphasize the complex mechanism by which MANF confers cardioprotection, involving not only the inhibition of UPR signaling but also the modulation of the JAK1/STAT1/NF-κB signaling pathway. Taken together, these discoveries offer valuable insights into the role of MANF in heart disease and provide important information for the development of novel strategies for heart disease treatment in the future.

Reperfusion or the rapid restoration of blood flow is essential for myocardial survival but can also lead to myocardial injury and ER stress. Research has shown that injecting rMANF can reduce I/R injury and identifying a key motif in MANF that regulates its secretion and transport suggests it could be a potential therapeutic target. Screening for medications that modulate MANF may offer treatment options for ER stress-related diseases. Further studies, including using genetically modified animal models with MANF overexpression, could confirm MANF as a valuable therapeutic target. Yet, it is important to note that the pharmacokinetics and pharmacodynamics of rMANF are not well-defined, and issues like instability and inactivation are common with recombinant proteins. Improvements are necessary to enhance the pharmacokinetic and pharmacodynamic properties of MANF for better efficacy in clinical applications, paving the way for effective treatment strategies for ER stress-related diseases.

## Data Availability

No datasets were generated or analysed during the current study.
